# Supplementary material for: Explainable emphysema detection on chest radiographs with deep learning
Source: PLoS One. 2022 Jul 28;17(7):e0267539. doi: 10.1371/journal.pone.0267539 (PMC9333227; doi:10.1371/journal.pone.0267539)

Supporting information

S1 Table. Preprocessing and data augmentation steps. Image preprocessing and augmentation steps are provided in the following table. Steps are applied consecutively. 1-4 and 11-12 are applied to all images and steps 5-10 are applied only for the training images to prevent overfitting.

Step	Method
**Image Preprocessing Methods (All images, before training)**	
1	Read DICOM image
2	Clip values above 99th percentile
3	Resize the longer side to 512, preserving the aspect ratio
4	Convert to 8-bit PNG files
**Data Augmentation Methods (during training only)**	
5	Load a batch of images
6	Uniformly sample an aspect ratio from [0.8, 1]:[0.8, 1] and scale it to image size
7	Randomly crop the image to the aspect ratio
8	Randomly flip the laterals left-right
9	Random scale [0.75, 1.25] and shift [-64, 64] (uniform sampling) pixel values
10	Clip pixel values outside [0, 255]
**Standardization Methods (during training, validation, test)**	
11	Histogram equalization
12	Resize to 512 by 512

S2 Table. Training Settings. Training setting for all models used in this study. These settings are chosen heuristically based on experiments with validation data.

**Setting**	**Value**
Framework	Tensorflow [35]
Model	ResNet-18 [29] from Classification models Zoo [36]
Weight Initialization	ImageNet pretrained weights
Cyclical Learning Rate Stage 1	[0.01, 0.001]
Cyclical Learning Rate Stage 2	[0.001, 0.0001]
Cyclical Learning Rate Stage 3	[0.0001, 0.00001]
Cyclical Learning Rate Step Size	2.5 epochs
Momentum	0.95
Nesterov	True
Early Stopping Condition	No improvement in validation cross-entropy
Early Stopping Epochs	10
Weight Regularizer	L2
Weight Regularization Coefficient	0.00003
Batch Size	16
Input Image Size	512 by 512

S3 Fig. Sign ROC curves with R1 as reference standard ROC curves of the emphysema sign models for each sign using the annotations of R1. Sensitivity and specificity point for R2 is provided for comparison. The 95% confidence intervals and error bars are calculated by bootstrapping.

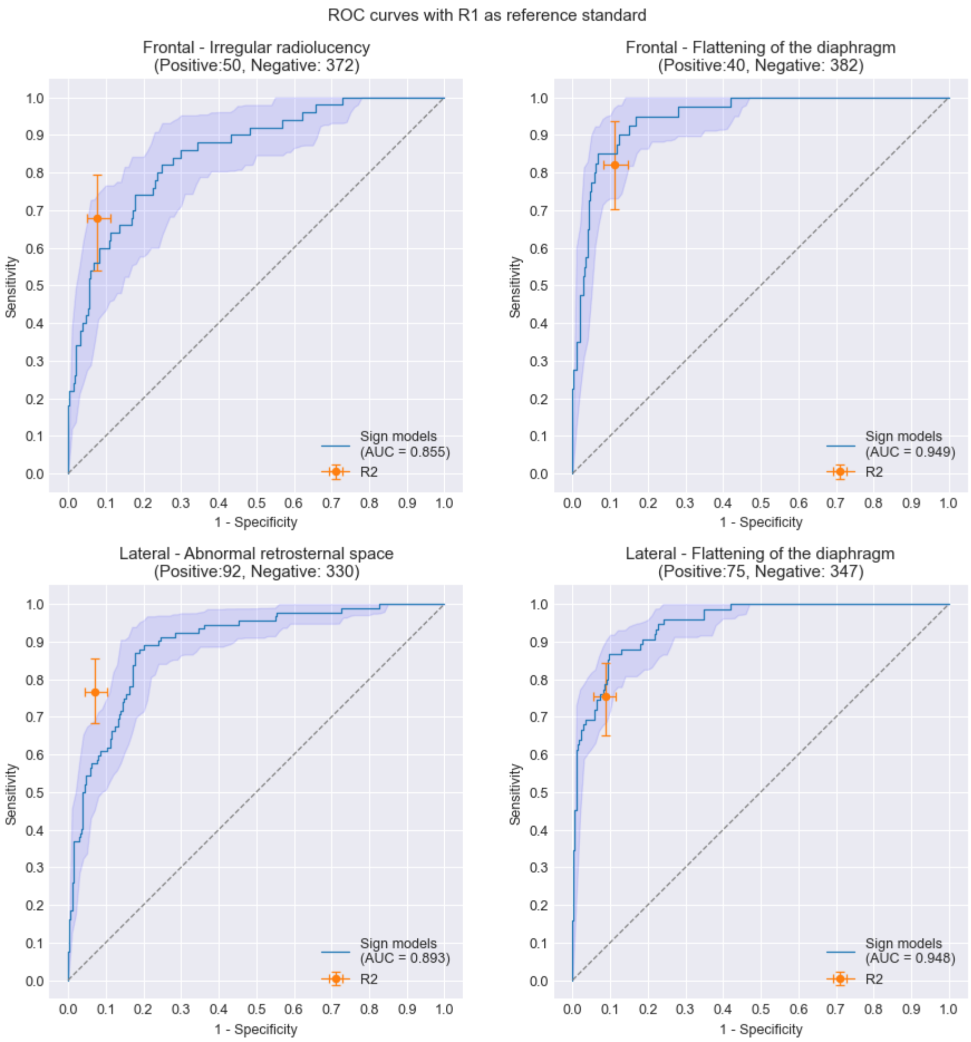

S4 Fig. Sign ROC curves with R2 as reference standard ROC curves of the emphysema sign models for each sign using the annotations of R2. Sensitivity and specificity point for R1 is provided for comparison. The 95% confidence intervals and error bars are calculated by bootstrapping.

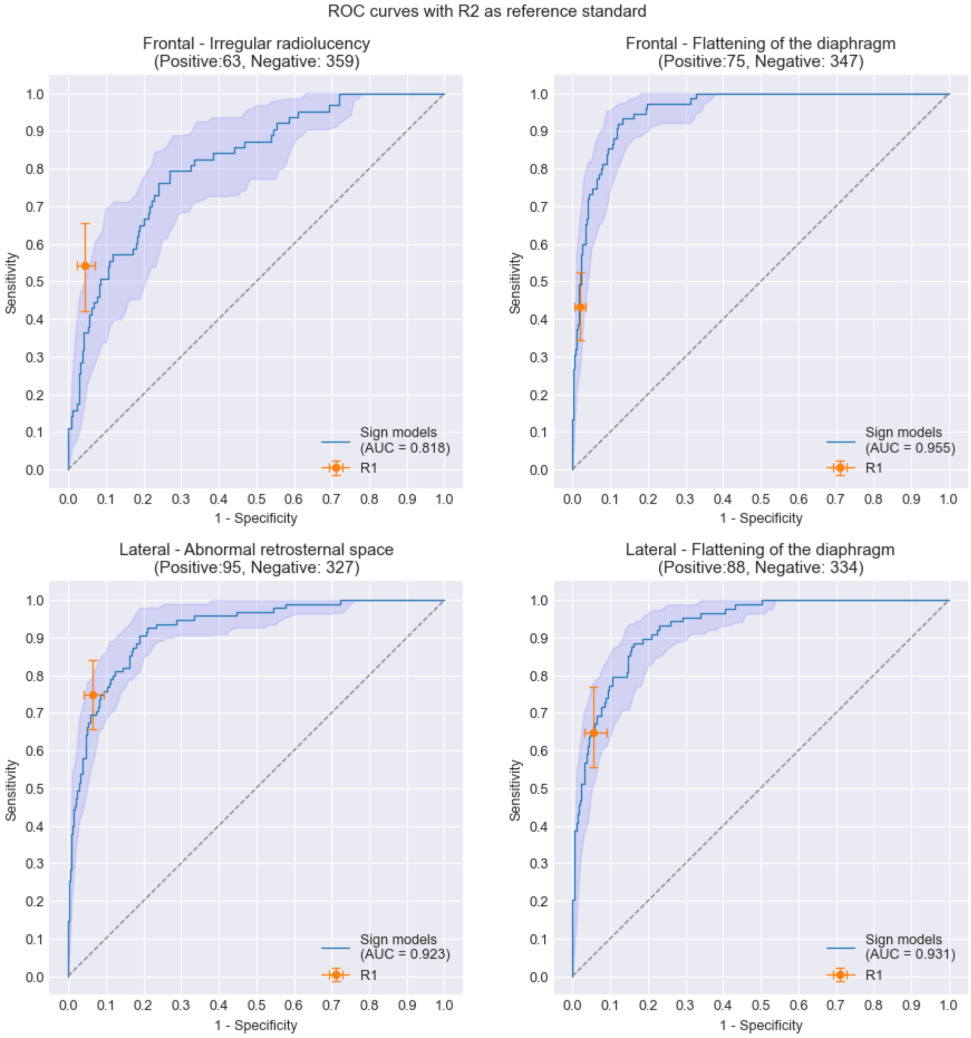

S5 Fig. Sign ROC curves on agreed upon annotations ROC curves of the emphysema sign models for each sign using the agreed upon annotations. The 95% confidence intervals are calculated by bootstrapping.

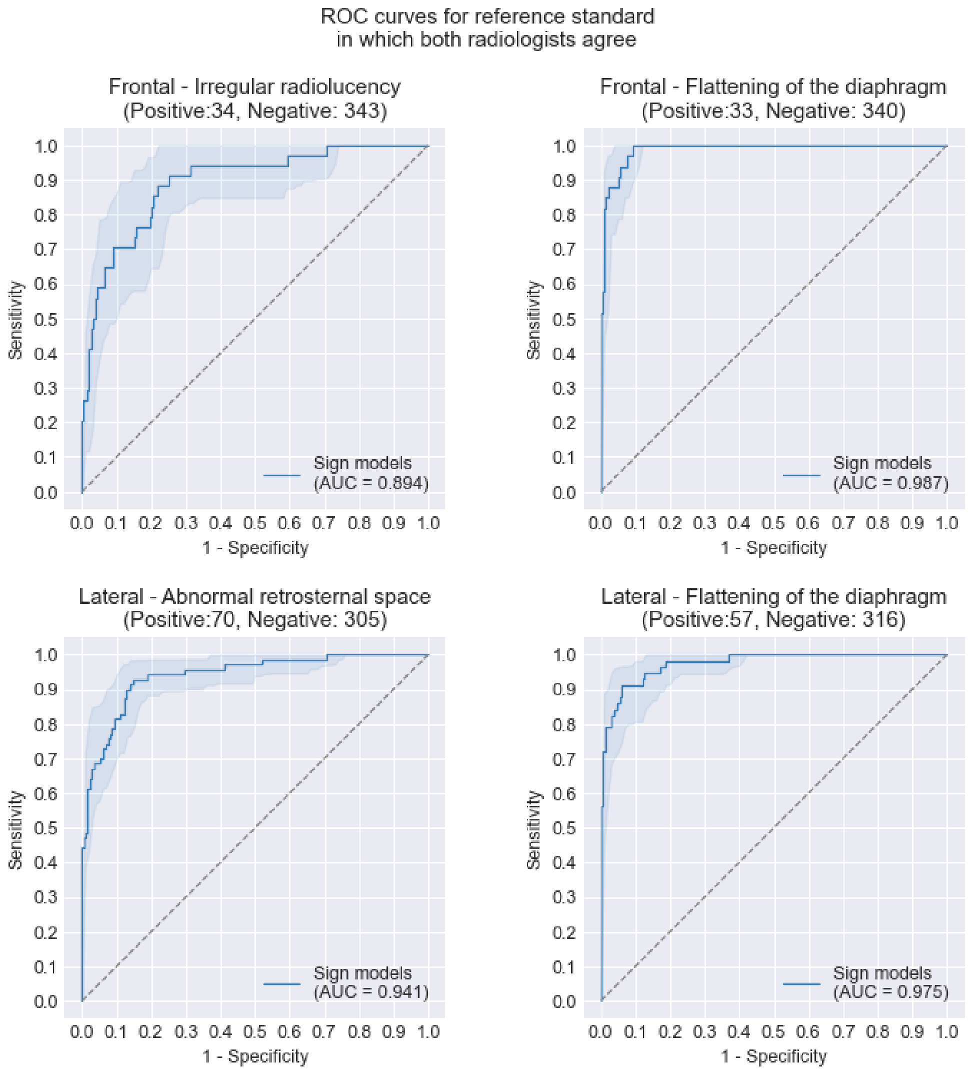

Supplement: S1 File — Collection of all supporting tables and figures. Including S1, S2 Tables, S3-S5 Figs. (PDF) [file pone.0267539.s001.pdf]
